# Exploring the Relationship between *CLPTM1L*-MS2 Variants and Susceptibility to Bladder Cancer

**DOI:** 10.3390/genes15010050

**Published:** 2023-12-28

**Authors:** Mi-So Jeong, Jeong-Yeon Mun, Gi-Eun Yang, Min-Hye Kim, Sang-Yeop Lee, Yung Hyun Choi, Heui Soo Kim, Jong-Kil Nam, Tae Nam Kim, Sun-Hee Leem

**Affiliations:** 1 Department of Biomedical Sciences, Dong-A University, Busan 49315, Republic of Korea; dkwl523@hanmail.net (M.-S.J.); jeongyeonmun90@gmail.com (J.-Y.M.); boos9063@naver.com (G.-E.Y.); kmhmary93@naver.com (M.-H.K.); 2 Research Center, Dongnam Institute of Radiological & Medical Sciences (DIRAMS), Busan 46033, Republic of Korea; 3 Department of Health Sciences, The Graduated of Dong-A University, Busan 49315, Republic of Korea; 4 Research Center for Bioconvergence Analysis, Korea Basic Science Institute, Ochang 28119, Republic of Korea; yopi@kbsi.re.kr; 5 Department of Biochemistry, College of Oriental Medicine, Anti-Aging Research Center, Dong-eui University, Busan 47227, Republic of Korea; choiyh@deu.ac.kr; 6 Department of Biological Sciences, College of Natural Sciences, Pusan National University, Busan 46241, Republic of Korea; khs307@pusan.ac.kr; 7 Department of Urology, Pusan National University Yangsan Hospital, Pusan National University School of Medicine, Research Institute for Convergence of Biomedical Science and Technology, Yangsan 50612, Republic of Korea; tuff-kil@hanmail.net; 8 Department of Urology, Pusan National University Hospital, Pusan National University School of Medicine, Biomedical Research Institute and Pusan National University Hospital, Busan 49241, Republic of Korea

**Keywords:** *CLPTM1L*, bladder cancer, VNTR polymorphism, cancer susceptibility

## Abstract

*CLPTM1L* (Cleft Lip and Palate Transmembrane Protein 1-Like) has previously been implicated in tumorigenesis and drug resistance in cancer. However, the genetic link between *CLPTM1L* and bladder cancer remains uncertain. In this study, we investigated the genetic association of variable number of tandem repeats (VNTR; minisatellites, MS) regions within *CLPTM1L* with bladder cancer. We identified four *CLPTM1L*-MS regions (MS1~MS4) located in intron regions. To evaluate the VNTR polymorphic alleles, we analyzed 441 cancer-free controls and 181 bladder cancer patients. Our analysis revealed a higher frequency of specific repeat sizes within the MS2 region in bladder cancer cases compared to controls. Notably, 25 and 27 repeats were exclusively present in the bladder cancer group. Moreover, rare alleles within the medium-length repeat range (25–29 repeats) were associated with an elevated bladder cancer risk (odds ratio [OR] = 5.78, 95% confidence interval [CI]: 1.49–22.47, *p* = 0.004). We confirmed that all MS regions followed Mendelian inheritance, and demonstrated that MS2 alleles increased *CLPTM1L* promoter activity in the UM-UC3 bladder cancer cells through a luciferase assay. Our findings propose the utility of *CLPTM1L*-MS regions as DNA typing markers, particularly highlighting the potential of middle-length rare alleles within *CLPTM1L*-MS2 as predictive markers for bladder cancer risk.

## 1. Introduction

Bladder cancer is a prevalent and significant health issue, particularly affecting men, with high incidence and mortality rates [1]. Non-muscle invasive bladder cancer (NMIBC) constitutes the majority of cases, and emphasizes the importance of primary prevention due to its high recurrence rates [2]. While tobacco smoking is a major risk factor, genetic predisposition also plays a role in bladder cancer [3]. Genome-wide association studies (GWAS) have identified multiple susceptibility loci, revealing novel genes involved in tumor development [4].

Among these genes, *CLPTM1L*, located in the chromosome 5p15.33 region, was initially recognized for its high expression in cisplatin-resistant ovarian cancer cells [5]. *CLPTM1L* has been implicated in chemoresistance against anticancer drugs such as cisplatin and camptothecin [6,7]. The TERT-*CLPTM1L* region has been extensively studied for single nucleotide polymorphisms (SNPs) and their association with various cancers, including bladder, breast, lung, prostate, and pancreatic cancer [8,9,10,11]. The rs401681 (C > T) SNP, located in intron 13 of *CLPTM1L*, is particularly linked to bladder cancer susceptibility [12]. Notably, this association is more prominent in Asian populations than in Caucasians [13]. While the VNTR polymorphism of TERT has been investigated in relation to cancer susceptibility [14,15,16,17], the VNTR polymorphism of *CLPTM1L* remains unexplored.

In this study, we characterized the VNTR region within the *CLPTM1L* gene through structural and sequence analysis. We confirmed the presence of VNTR polymorphism in *CLPTM1L* using genomic DNA obtained from cancer-free controls and bladder cancer patients. By comparing the incidence of each allele between the control and patient groups, we determined the association between VNTR polymorphism and bladder cancer risk. Additionally, we conducted a luciferase assay to assess the impact of bladder cancer-associated VNTR alleles on *CLPTM1L* promoter activity. Furthermore, we examined the vertical transmission of this VNTR region across generations using a family DNA sample.

The findings of this study suggest that the *CLPTM1L* VNTR region may be associated with bladder cancer risk through the modulation of *CLPTM1L* gene expression. These insights contribute to our understanding of the genetic basis of bladder cancer susceptibility.

## 2. Materials and Methods

### 2.1. Structural Analysis and Primer Construction for VNTR Regions of CLPTM1L

NCBI (http://www.ncbi.nlm.nih.gov/gene) was used to confirm the location of *CLPTM1L* on chromosome 5, the composition of its exons and introns, and its DNA sequence (NC_000005.10:c1345099-1317752 Homo sapiens chromosome 5, GRCh38.p13 Primary Assembly). The VNTR regions were identified using the Tandem Repeats Finder (https://tandem.bu.edu/trf/trf.html) [18]. VNTR regions were selected based on the length of the repeat unit, which had to be between 10 and 100 bp and scored > 500 in the program algorithm. Primers for the selected VNTR regions were designed using primer 3 (http://bioinfo.ut.ee/primer3-0.4.0/) and confirmed using Primer-BLAST at NCBI (http://www.ncbi.nlm.nih.gov/tools/primer-blast/). The UCSC in silico PCR (https://genome.ucsc.edu/cgi-bin/hgPcr) was used to confirm that these primers amplified the correct PCR products in the *CLPTM1L* gene. The sequences of the primer pairs used for amplification are listed in Table 1.

### 2.2. Population for Case–Control Study

To confirm the polymorphism in the *CLPTM1L* VNTR regions, we compared genomic DNA from 441 cancer-free male controls to samples obtained from 181 male patients with bladder cancer, while eliminating gender differences. The controls had a similar age distribution to the bladder cancer patients, with an average age of 65.25 years (range: 50–88) for controls and 66.52 years (range: 50–90) for patients. Additionally, genomic DNA samples were obtained from eight multigenerational family groups, including six two-generation families and two three-generation families. For conducting PCR experiments, we utilized genomic DNA that was isolated in a previous study [19,20,21].

### 2.3. Polymerase Chain Reaction (PCR) Amplification of the VNTR Regions

Genomic DNA samples were used as templates for PCR amplification of each VNTR region. The PCR mixture contained 100 ng of genomic DNA, 10 pmol of primer pairs, and Taq polymerase. We used EmeraldAmp PCR Master Mix (Takara Bio, Inc., Tokyo, Japan). The PCR conditions for each VNTR region were as follows: *CLPTM1L*-MS1 and -MS2 underwent an initial denaturation at 94 °C for 2 min, followed by 30 cycles of 30 s at 94 °C and 2 min at 68 °C, and a final extension for 10 min at 72 °C; *CLPTM1L*-MS3 underwent an initial denaturation at 94 °C for 2 min, followed by 33 cycles of 30 s at 94 °C, 20 s at 68.4 °C, and 3 min at 72 °C, and a final extension for 7 min at 72 °C; *CLPTM1L*-MS4 underwent an initial denaturation at 94 °C for 2 min, followed by 30 cycles of 30 s at 94 °C, 20 s at 65.5 °C, and 2 min at 72 °C, and a final extension for 7 min at 72 °C. PCR amplification was performed using a 9700 Thermal Cycler (Perkin-Elmer, Inc., Waltham, MA, USA).

### 2.4. Analysis of VNTR Polymorphism through Electrophoresis

The VNTR regions were analyzed using gel electrophoresis (1 V/cm) in 1× TAE buffer through a 0.7–2.0% agarose gel. The electrophoresis conditions were set differently depending on the repeat unit and length of the PCR product for each VNTR region. For MS1, a 2% SeaKem ^®^ LE agarose gel (Lonza, Rockland, ME, USA) was run at 120 V for 6 h. MS2 was run on a 1.5% LE agarose gel at 80 V for 14 h, and MS4 was run on a 2% LE agarose gel at 120 V for 4 h. A 100 bp DNA ladder (ELPIS-Biotech, Daejeon, Republic of Korea) was used as a marker for the electrophoresis of these three regions. The length of the repeat unit for MS3 was 29 bp. Since the length of the PCR product was about 3 kb, the PCR product was run on a 0.7% LE agarose gel at 60 V for 20 h. In this case, a 1 kb DNA ladder (Invitrogen, Carlsbad, CA, USA) was used as a marker.

### 2.5. Construction of CLPTM1L Luciferase Reporter Vector Containing CLPTM1L-MS2

The promoter region sequence of *CLPTM1L* was obtained from the NCBI website and confirmed (NC_000005.10:c1342099-1317752 Homo sapiens chromosome 5, GRCh38.p13 Primary Assembly). To construct the *CLPTM1L* luciferase reporter vector, the pGL3-Basic vector (Promega, Madison, WI, USA) and a fragment of the *CLPTM1L* promoter (3 kb region upstream of the first ATG of *CLPTM1L*) were digested by restriction enzymes (NheI/XhoI, NEB, New England Biolabs, Inc., Hitchin, UK). The resulting fragments were purified using a gel extraction kit (QIAquick gel extraction kit, Qiagen, Hilden, Germany), and then ligated using T4 ligase (NEB, Inc., UK). Common alleles (TR23) and rare alleles (TR17, TR23, TR25, TR27, TR29, and TR31) were inserted into the HpaI/BamHI digested site of the *CLPTM1L* promoter plasmid. The constructed vectors were confirmed via DNA sequencing.

### 2.6. Cell Culture and Luciferase Assay

To examine effect of *CLPTM1L*-MS2 on *CLPTM1L* expression, 293T (a human embryonic kidney cell line) and UM-UC3 (bladder cancer cell line) were used. Cells (5 × 10^4^ per well) were seeded in 24-well plates and cultured for 24 h. Cells were transfected with pGL3-Basic *CLPTM1L* promoter plasmid and *CLPTM1L*-MS2 containing promoter plasmids (0.3 μg per well) using jetPrime transfection reagent (Polyplus, New York, NY, USA). *CLPTM1L* promoter activities were measured using the dual-luciferase reporter assay system (Promega, Madison, WI, USA) 48 h after cell transfection. Firefly luciferase activities were analyzed based on Renilla luciferase activities and represented relative luciferase units, which indicate promoter activity.

### 2.7. Statistical Analysis

The degree of polymorphism (heterozygosity) ranges from 0 to 1, which increases with the number of alleles. The probability that two randomly selected alleles differ at a given locus was calculated through the formula as previously described [22]. A regression analysis was performed to determine the ORs (odd ratios) for the association between controls and cases groups. ORs were estimated using the natural logarithm and its standard error. Where appropriate, we used the chi-squared test with one degree of freedom to evaluate differences between groups. Significant differences were determined using a 95% confidence interval (CI). All of the tests were two-sided, with *p* < 0.05 considered statistically significant. To calculate the chi-squared values, a statistical analysis was performed using MS Excel with CHITEST and R statistical software (https://www.socscistatistics.com/tests/fisher/default2.aspx) using the chi-squared test function.

## 3. Results

### 3.1. Selection of CLPTM1L VNTR Region

**Figure 1:**
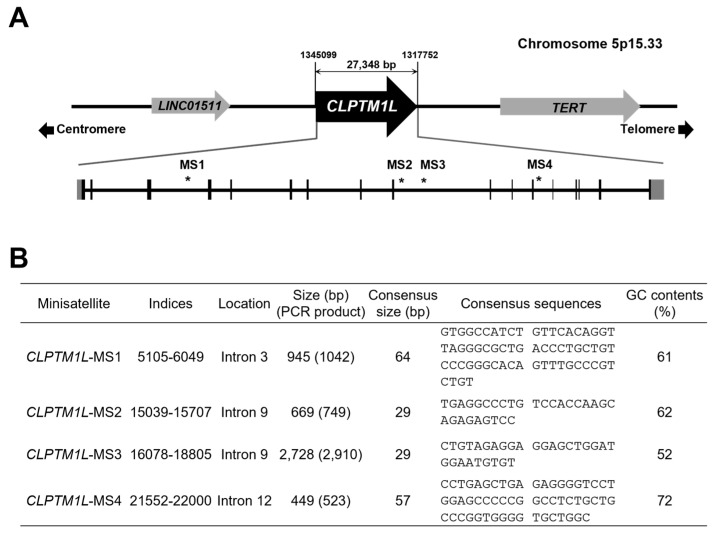
The information of VNTR in *CLPTM1L*. (**A**) The structure of genomic region of *CLPTM1L*. It is predicted to contain 17 exons (represented by black boxes) that encode *CLPTM1L*. The approximate locations of the VNTRs (MS1, MS2, MS3, and MS4) are indicated by asterisks and numbers on the diagram. (**B**) The table provides information on the location, PCR product sizes, consensus repeat unit sizes, consensus sequences of the repeat unit, and GC contents of the four VNTR regions. VNTRs, variable number of tandem repeats. Asterisks indicate the location of each VNTR region.

The genomic location of the *CLPTM1L* gene on chromosome 5p15.33 was identified, along with neighboring genes and the structure of its exons and introns (Figure 1A). *CLPTM1L* spans approximately 27 kb, and comprises 17 exons and 16 introns. The gene is oriented towards the telomeres, and is positioned upstream of the TERT gene and downstream of the LINC01511 gene. Utilizing the Tandem Repeat Finder program, four VNTR regions (*CLPTM1L*-MS1~MS4) within *CLPTM1L* were detected (algorithm scores > 500). All VNTR regions were found within the intronic regions. The size of each VNTR, the anticipated PCR product size, consensus repeat size, and sequences were determined based on the NCBI sequence (Figure 1B).

### 3.2. Polymorphic Analysis of CLPTM1L VNTR Regions

**Figure 2:**
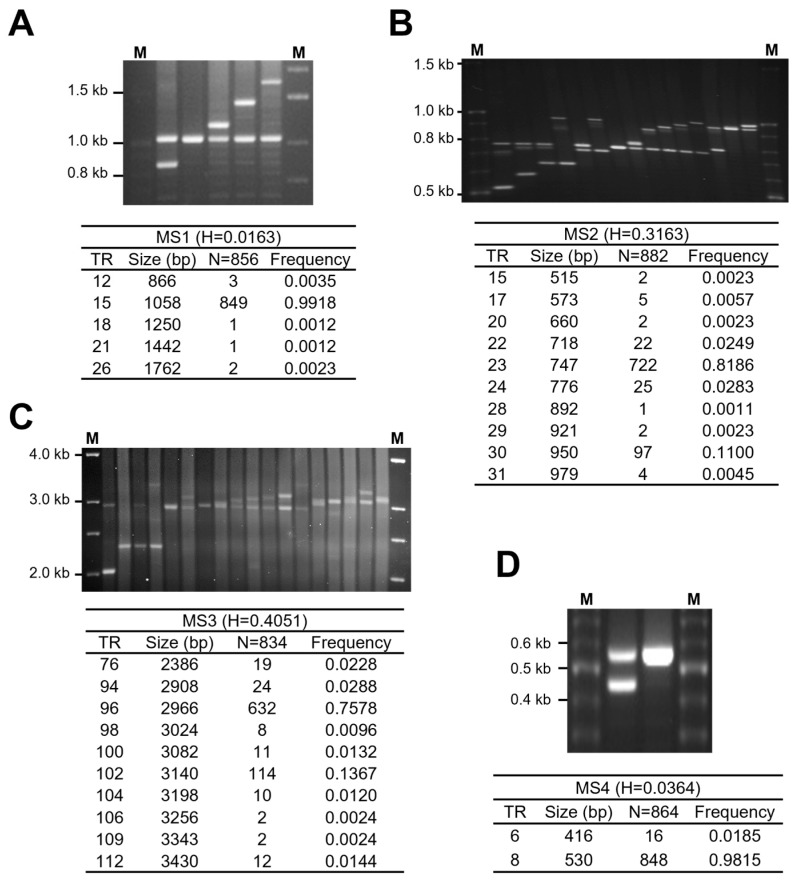
The allelic frequencies and electrophoretic patterns of *CLPTM1L* VNTRs in cancer-free controls. The allelic patterns of each VNTR are shown in the upper part (electrophoretic patterns) of each panel (**A**–**D**; *CLPTM1L*-MS1 to MS4). The lower part of each panel indicates the allelic frequency, size of PCR products, and repeat number. VNTRs were amplified from genomic DNA of cancer-free controls using PCR techniques with each primer (see the Materials and Methods section). The table below the figure shows the number of alleles of four polymorphic VNTRs: 5 alleles of MS1 (**A**), 10 alleles of MS2 (**B**), 10 alleles of MS3 (**C**), and 2 alleles of MS4 (**D**). Size markers are given in bp (100 bp or 1 kb size markers). H represents the heterozygosity of each VNTR in cancer-free controls.

Genomic DNA samples obtained from cancer-free controls were utilized to confirm the presence of polymorphisms in each VNTR region (Figure 2). Given that bladder cancer incidence is significantly higher in men than in women, male samples were used as cancer-free controls (Table 2). Initially, 100 cancer-free control samples were analyzed to confirm the polymorphic nature of each VNTR region. Subsequently, all regions were determined to be polymorphic, and were further analyzed using additional samples.

*CLPTM1L*-MS1, located in the intron 3 region, exhibited polymorphism with 5 alleles ranging from 12 to 26 repeats. The most common allele consisted of 15 repeats (99.2%). This region demonstrated the lowest heterozygosity among the four regions, with a value of 0.0163 (Figure 2A). *CLPTM1L*-MS2 and MS3 were identified in the intron 9 region. *CLPTM1L*-MS2 displayed 10 alleles varying between 15 and 31 repeats (Figure 2B), with the most common allele containing 23 repeats (81.9%). In the case of *CLPTM1L*-MS3, 10 alleles were observed, ranging from 76 to 112 repeats (Figure 2C). Notably, the MS3 region exhibited the highest heterozygosity among the four VNTR regions, with a value of 0.4051. In the intron 12 region, *CLPTM1L*-MS4 comprised 2 alleles with lengths of 416 and 530 base pairs (bp) consisting of 6 and 8 repeats, respectively. Only the common allele had a frequency greater than 1%, with the most prevalent allele containing 8 repeats (Figure 2D).

### 3.3. Mendelian Inheritance of CLPTM1L VNTR

**Figure 3:**
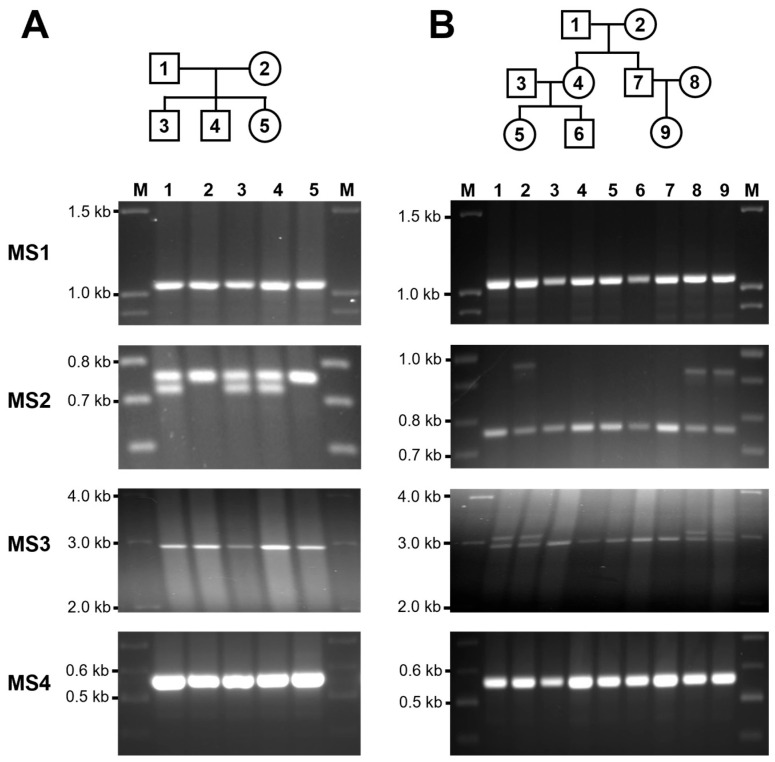
Meiotic inheritance of *CLPTM1L* VNTRs in a two- and a three-generation family. (**A**) The first generation (lanes 1 and 2; father and mother, respectively) and the second generation (lanes 3, 4 and 5; children of parents 1 and 2). (**B**) The first generation (lanes 1 and 2; parent of lane 4 and 7), second generation (lanes 4 and 7; children of parents 1 and 2), and third generation (lanes 5 and 6; children of parents 3 and 4, lanes 9; children of parents 7 and 8) are shown. The size marker is represented by M.

An analysis of the inheritance patterns of the VNTR regions in *CLPTM1L* was conducted using family groups comprising two and three generations (Figure 3). Genomic DNA samples previously obtained from each family member were utilized [18,19]. The confirmation of each VNTR region was performed in eight families. Meiotic transfer of all VNTRs was traced from parents to offspring, and hereditary segregation was observed in both two-generation (Figure 3A) and three-generation (Figure 3B) families. The segregation patterns within each family indicated that these VNTRs were transmitted through meiosis in accordance with Mendelian inheritance principles (i.e., each child inherited one VNTR allele from each parent; Figure 3).

### 3.4. Association of CLPTM1L VNTR with Allelic Variation and Bladder Cancer

A case–control study was conducted to investigate the association between VNTR alleles and bladder cancer. The study included 441 cancer-free control subjects and 181 bladder cancer patients. The frequencies of VNTR alleles in the *CLPTM1L*-MS1, MS3, and MS4 regions were compared between the two groups (Supplementary Table S1). The results indicated no significant differences in the frequencies of these alleles between the control subjects and bladder cancer patients. However, it was observed that the *CLPTM1L*-MS3 region had 128 repeats exclusively in the cancer patient group. Nonetheless, even when considering the genotypes of these three VNTR regions, no significant association with bladder cancer risk was found (Supplementary Table S2).

**Figure 4:**
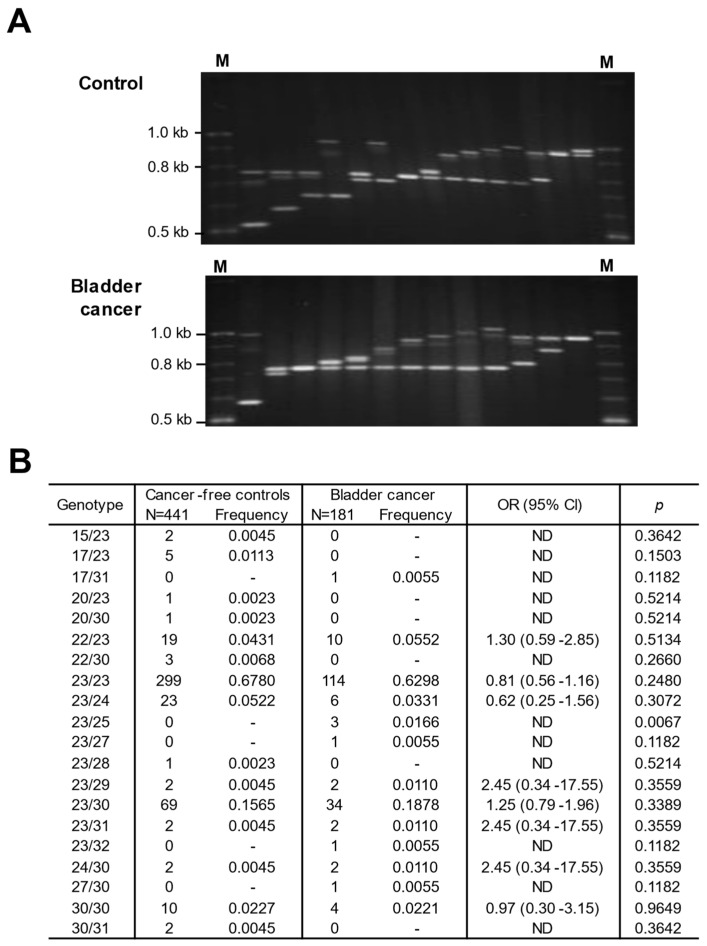
Comparison of genotypes of *CLPTM1L*-MS2 between cancer-free controls and bladder cancer patients. (**A**) Electrophoretic pattern of *CLPTM1L*-MS2 in cancer-free controls (upper panel) and patients with bladder cancer (lower panel). M indicates the size marker. (**B**) Frequency of genotypes between controls and bladder cancer cases. N represents the total number of samples used to genotype the *CLPTM1L*-MS2.

Further analysis focused on the *CLPTM1L*-MS2 region, comparing the allele frequencies between control subjects and bladder cancer patients (Table 3 and Figure 4A). The common alleles in this region were 22, 23, 24, and 30 repeats, with 23 repeats being the most prevalent. Rare alleles with a frequency of less than 1% were categorized based on their length (Table 3). Significantly different frequencies were observed for the middle-length rare alleles with 25 repeats (*p* = 0.0068) and 27 repeats (*p* = 0.0272). Moreover, the group of middle-length rare alleles (consisting of 25, 27, 28, and 29 repeats) showed a significant difference between cancer-free controls and bladder cancer patients (OR = 5.78, 95% CI: 1.49–22.47, *p* = 0.004).

In the investigation of the *CLPTM1L*-MS2 genotype within the context of bladder cancer, the presence of one middle-length rare allele (MR/C) in individuals (cases:controls = 3.67%:0.68%) was associated with an increased risk of bladder cancer, as indicated by an odds ratio of 5.87 (95% confidence interval: 1.5–22.97; *p* = 0.0041) (Table 4; Figure 4). Specifically, the 23/25 genotype was found to be more prevalent in bladder cancer patients compared to the control group, and this disparity demonstrated statistical significance (*p* = 0.0067) (Table 4; Figure 4B).

### 3.5. The Potential Effect of CLPTM1L-MS2 on the Gene Expression

**Figure 5:**
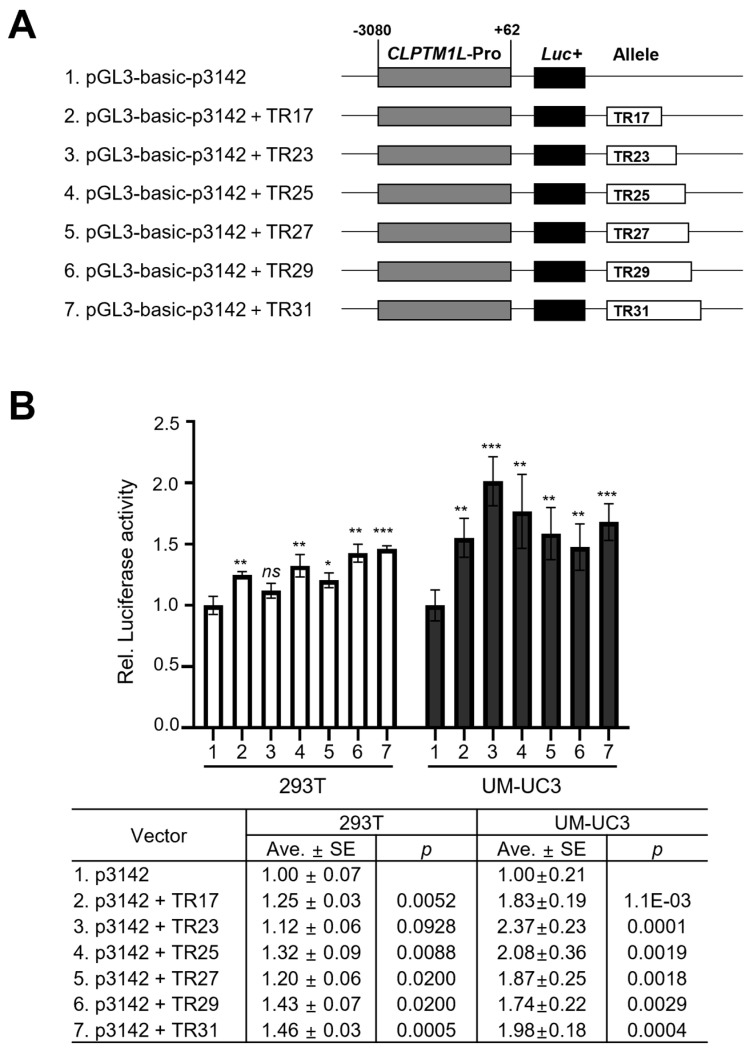
Effect of alleles of *CLPTM1L*-MS2 in *CLPTM1L* promoter luciferase constructs. (**A**) The structure of the p3142 (#1) and TR reporter constructs. The gray square indicates the *CLPTM1L* promoter region. The black square represents the open reading frame of luciferase. The open squares represent the VNTR polymorphic regions of *CLPTM1L*-MS2. Six different sizes of TR (17–31 repeats) were inserted into the p3142 plasmid (#2–#7). (**B**) The effects of VNTR polymorphism on *CLPTM1L* gene expression in the luciferase reporter system. Seven different plasmids were transfected into two different cell lines (293T, embryonic kidney and UM-UC3, bladder cancer) for 48 h. (* *p* < 0.05; ** *p* < 0.01; *** *p* < 0.001).

To assess the potential effect of *CLPTM1L*-MS2 on the expression of *CLPTM1L*, alleles of the MS2 region were inserted into the *CLPTM1L* promoter vector (Figure 5A). Luciferase assays were conducted using the embryonic kidney 293T cell line and the bladder cancer UM-UC3 cell line. The results demonstrated that the promoter activity of *CLPTM1L* was enhanced in the vector containing the *CLPTM1L*-MS2 alleles compared to the *CLPTM1L* promoter vector alone. Additionally, the promoter activity of the vector containing the *CLPTM1L*-MS2 allele was significantly higher in the UM-UC3 cell line compared to the 293T cell line (Figure 5B). However, no influence on gene expression was observed based on the length of the *CLPTM1L*-MS2 alleles. These findings suggest that the *CLPTM1L*-MS2 region may modulate the expression of *CLPTM1L* by augmenting the activity of the *CLPTM1L* promoter.

Previous studies have provided evidence supporting the influence of VNTR regions located within introns on gene expression [23,24,25]. Consistent with these findings, the present study also recognized VNTR regions as potential regulators of gene expression. Therefore, a specific analysis was conducted to examine the composition of the 23 most frequently observed repeat alleles, aiming to identify characteristics of the repeat units within the *CLPTM1L*-MS2 region that might impact gene expression (Supplementary Table S3). The 23-repeat allele was determined to consist of 8 repeat units, exhibiting a range of homology from 78% to 97% between repeat sequences (Supplementary Table S4). Furthermore, in order to gain insights into the functional properties associated with gene regulation within these sequences, the Transfac software (MATCHTM Publication Version 1.0; http://www.gene-regulation.com/pub/databases.html) was employed to analyze the repetitive regions. Our analysis revealed several potential binding sites for transcription factors such as GATA-1 and NF-1, as illustrated in Supplementary Figure S1.

## 4. Discussion

In this study, we conducted a genomic sequence analysis of the *CLPTM1L* gene and identified four novel VNTR regions (*CLPTM1L*-MS1~MS4). These regions exhibited no significant similarity to any previously reported VNTR regions when assessed using the BLASTN program. Thus, all VNTR regions chosen for this study are exclusive to *CLPTM1L*, and their characteristics may be linked to the gene’s functionality. These VNTRs were situated within the intron region, and exhibited polymorphism with more than two alleles. The number of repetitions within the nucleotide sequence unit varied between the two alleles of an individual and also displayed variations across individuals, rendering them valuable as markers for personal identification [26]. It was confirmed that the inheritance pattern of alleles for each VNTR region of *CLPTM1L* followed Mendelian inheritance from the parental generation to the offspring generation. Consequently, all *CLPTM1L*-VNTR regions can serve as markers for paternity identification and DNA typing.

Previous studies have suggested that polymorphisms in the VNTR regions of certain genes are associated with the risk for various types of cancer [27,28]. In this study, we focused on investigating polymorphisms within the *CLPTM1L* VNTR region, which belong to a cancer-associated region containing the TERT gene located at the p-arm end of chromosome 5 [29]. In our case–control study, significant differences in allele frequencies were observed exclusively in the *CLPTM1L*-MS2 region among the four regions analyzed. In particular, the frequency of rare alleles of intermediate length was found to be higher in bladder cancer patients compared to controls, especially within this region. Additionally, individuals carrying the rare allele of intermediate length showed a 5.87-fold increased risk of developing bladder cancer (95% CI: 1.5–22.97, *p* = 0.004), which demonstrates statistical significance. This study represents the first comprehensive characterization of the *CLPTM1L* VNTR regions, and based on our findings, it may serve as an index for assessing the risk of bladder cancer.

The expression of *CLPTM1L* was observed to be elevated in lung adenocarcinoma and pancreatic cancer tissues compared to adjacent normal tissues, and poor clinical outcomes were associated with high *CLPTM1L* expression in patients with pancreatic and lung cancer [30,31]. VNTR sequences can serve as regulatory elements, influencing both transcriptional and translational processes. When located within the exon region of a gene, VNTRs can impact protein function, while in the intron region, they can affect gene expression [23,24,25]. In this study, the impact of the *CLPTM1L*-MS2 region, which exhibited a statistically significant association with bladder cancer risk on the promoter activity of *CLPTM1L*, was validated through luciferase analysis. Although no variation was observed based on allele length, it was confirmed that *CLPTM1L*-MS2 modulates *CLPTM1L* gene expression by demonstrating that different alleles exert an influence on *CLPTM1L* promoter activity. This effect may be attributed to the binding of transcription factors to the *CLPTM1L*-MS2 region.

The role of transcription factors in the development of bladder cancer has been reported [32], and in this study, the putative binding sites of several transcription factors were identified within the VNTR regions (Supplementary Figure S1). The glucocorticoid receptor (NR3C1, GR), a nuclear hormone receptor, regulates the expression of genes associated with inflammation and plays a role in suppressing cell proliferation in bladder cancer [33,34]. AP-2alpha is highly expressed in basal-squamous bladder cancer cell lines and correlates with increased distant recurrence and lymph node metastasis [35]. The androgen receptor (AR), a ligand-activated transcription factor, controls genes that are crucial for male sexual differentiation and development, and its involvement in bladder cancer carcinogenesis has been reported [36,37]. General transcription factor IIi (GTF2I, TFII-I) is a multifunctional transcription factor that regulates the expressions of genes involved in cell proliferation, angiogenesis, and cellular stress response [38]. An elevated expression of nuclear factor I (NFI) transcription factor has been associated with tumor progression and drug resistance in bladder cancer [39,40]. Paired-box gene 5 (PAX5) expression in undifferentiated transitional cell carcinoma (TTC) of the bladder may contribute to pathogenesis by supporting cellular proliferation in the dedifferentiated state [41]. Mutation of the p53 gene and nuclear accumulation of the p53 protein are linked to bladder cancer grade and stage, playing a significant role in the progression of the disease [42]. TEAD (ETF) participates in tumor progression, including tumor development, drug resistance, epithelial–mesenchymal transition (EMT), and metastasis, by engaging various oncogenic signaling pathways such as Wnt, TGFβ, and Hippo signaling [43]. CCAAT enhancer-binding protein β (CEBPB) activity can be induced by oncogenic Ras, and contributes to Ras-mediated tumorigenesis and cell proliferation [44]. Neurofibromin 1 (NF-1) mutation is associated with an increased risk of various tumors, including melanoma and breast cancer [45]. Thyroid hormone receptor β (THRB, T3R-beta1) is frequently mutated and downregulated in human cancers such as breast, lung, and thyroid cancer, suggesting its potential role as a tumor suppressor [46]. GATA-binding protein 1 (GATA1) participates in tumor progression by activating JAG1/Notch and PI3K/AKT pathways in ovarian and colorectal cancer, respectively [47,48].

In summary, this study has identified four novel VNTR regions within the *CLPTM1L* gene. These regions exhibit uniqueness and polymorphism, making them valuable as markers for personal identification and DNA typing. Importantly, polymorphisms within the *CLPTM1L*-MS2 region are linked to an increased susceptibility to bladder cancer, particularly among individuals carrying the middle-length rare allele. The *CLPTM1L*-MS2 region has been found to influence the expression of the *CLPTM1L* gene, potentially mediated by the binding of transcription factors to this region. Elevated *CLPTM1L* expression is associated with heightened cancer malignancy and resistance to anticancer drugs. Notably, our study focuses on the relationship between *CLPTM1L* variants and the risk of developing bladder cancer, but is limited by the lack of analysis using clinical data from bladder cancer patients. Thus, we recommend further research to validate the link between *CLPTM1L* genetic variants and the progression and responsiveness to anticancer drugs by analyzing clinical data from bladder cancer patients.

## Figures and Tables

**Table 1:** Sequences of primer pairs.

Minisatellite	Forward Primer Sequence	Reverse Primer Sequence
MS1	CCCTCTCTGCTGGGCTCTCC	CTCCGCCTCGATCTGCTGTT
MS2	TGAACAAGTGGAGAGCAAGGAAA	ACATGGTGGGATTTCTCAAGCAC
MS3	GTCTCTGCAGTTGGTGGCCC	CTGAGAGCCGCCACCTCATG
MS4	CCACCAGGCTTCATGGGAGG	AGTGCTGAGCCTGGTTCTC

**Table 2:** Age distribution of controls and bladder cancer cases.

Characteristic		Cancer-Free Controls, N (%)	Bladder Cancer Cases, N (%)	*p*
Age	50–59	128 (29.02)	46 (25.41)	0.935
	60–69	164 (37.19)	68 (37.57)	
	70–79	127 (28.80)	56 (30.94)	
	≥80	22 (4.99)	11 (6.08)	
	Average	65.25	66.52	
	Median	65	67	
	N	441	181	

**Table 3:** Analysis of *CLPTM1L*-MS2 alleles between controls and bladder cancer cases.

TR Group	Repeats	Size (bp)	Cancer-Free Controls		Bladder Cancer		OR (95% CI)	*p*
			N = 882	Frequency	N = 362	Frequency		
SR	15	515	2	0.0023	0	-	ND	0.3645
	17	573	5	0.0057	1	0.0028	0.49 (0.06–4.17)	0.5020
	20	660	2	0.0023	0	-	ND	0.3645
	Total		9	0.0102	1	0.0028	0.27 (0.03–2.13)	0.1820
SC	22	718	22	0.0249	10	0.0276	1.11 (0.52–2.37)	0.7862
	23	747	722	0.8186	287	0.7928	0.85 (0.62–1.15)	0.2914
	24	776	25	0.0283	8	0.0221	0.77 (0.35–1.73)	0.5335
	Total		769	0.8719	305	0.8425	0.79 (0.56–1.11)	0.1710
MR	25	805	0	-	3	0.0083	ND	0.0068 *
	27	863	0	-	2	0.0055	ND	0.0272 *
	28	892	1	0.0011	0	-	ND	0.5216
	29	921	2	0.0023	2	0.0055	2.44 (0.34–17.42)	0.3567
	Total		3	0.0034	7	0.0110	5.78 (1.49–22.47)	0.0040 *
LC	30	950	97	0.1100	45	0.1243	1.15 (0.79–1.67)	0.4700
	Total		97	0.1156	45	0.1409	1.15 (0.79–1.67)	0.4700
LR	31	979	4	0.0045	3	0.0083	1.83 (0.41–8.24)	0.4216
	32	1008	0	-	1	0.0028	ND	0.1184
	Total		4	0.1202	4	0.1519	2.45 (0.61–9.86)	0.1920

SR (short rare alleles, ≥20 repeats); SC (short common alleles, 22~24 repeats); MR (middle rare alleles, 25~29 repeats); LC (long common allele, 30 repeat); LR (long rare alleles, ≤31 repeats); OR, odds ratio; CI, confidence internal; ND, not determined; * statistically significant (*p* < 0.05).

**Table 4:** Analysis of *CLPTM1L*-MS2 genotypes and risk of bladder cancer.

Genotype Group	Genotype	Cancer-Free Controls		Bladder Cancer		OR (95% CI)	*p*
		N = 441	Frequency	N = 181	Frequency		
SR/–	15/23	2	0.0045	0	-	ND	0.3642
	17/23	5	0.0113	0	-	ND	0.1503
	17/31	0	-	1	0.0055	ND	0.1182
	20/23	1	0.0023	0	-	ND	0.5214
	20/30	1	0.0023	0	-	ND	0.5214
	Total	9	0.0204	1	0.0055	0.27 (0.03–2.12)	0.1811
MR/–	23/25	0	-	3	0.0166	ND	0.0067 *
	23/27	0	-	1	0.0055	ND	0.1182
	23/28	1	0.0023	0	-	ND	0.5214
	23/29	2	0.0045	2	0.0110	2.45 (0.34–17.55)	0.3559
	27/30	0	-	1	0.0055	ND	0.1182
	Total	3	0.0068	7	0.0367	5.87 (1.50–22.97)	0.0041 *
LR/–	17/31	0	-	1	0.0055	ND	0.1182
	23/31	2	0.0045	2	0.0110	2.45 (0.34–17.55)	0.3559
	23/32	0	-	1	0.0055	ND	0.1182
	30/31	2	0.0045	0	-	ND	0.3642
	Total	4	0.0091	4	0.2210	2.47 (0.61–9.98)	0.1902

SR (short rare alleles, ≥20 repeats); MR (middle rare alleles, 25~29 repeats); LR (long rare alleles, ≤31 repeats); - (other alleles); OR, odds ratio; CI, confidence internal; ND, not determined; * statistically significant (*p* < 0.05).

## Data Availability

All data generated during this study are included in this published article and its supplementary information files.

## Supplementary Materials

The following supporting information can be downloaded at: https://www.mdpi.com/article/10.3390/genes15010050/s1, Table S1: Frequency of *CLPTM1L*-VNTR (MS1, MS3, MS4) alleles between controls and bladder cancer cases; Table S2: Frequency of *CLPTM1L*-VNTR (MS1, MS3, MS4) genotypes between controls and bladder cancer cases; Table S3: Composition of repeat units in most common alleles of *CLPTM1L*-MS2; Table S4: Analysis of identities between repeat units in *CLPTM1L*-MS2; Figure S1: Composition of putative transcriptional factors on most common alleles of *CLPTM1L*-MS2.

## References

[1] Sung H., Ferlay J., Siegel R.L., Laversanne M., Soerjomataram I., Jemal A., Bray F. (2021). Global Cancer Statistics 2020: GLOBOCAN Estimates of Incidence and Mortality Worldwide for 36 Cancers in 185 Countries. CA Cancer J. Clin..

[2] Al-Zalabani A.H., Stewart K.F., Wesselius A., Schols A.M., Zeegers M.P. (2016). Modifiable risk factors for the prevention of bladder cancer: A systematic review of meta-analyses. Eur. J. Epidemiol..

[3] Knowles M.A., Hurst C.D. (2015). Molecular biology of bladder cancer: New insights into pathogenesis and clinical diversity. Nat. Rev. Cancer.

[4] Dudek A.M., Grotenhuis A.J., Vermeulen S.H., Kiemeney L.A., Verhaegh G.W. (2013). Urinary bladder cancer susceptibility markers. What do we know about functional mechanisms?. Int. J. Mol. Sci..

[5] Yamamoto K., Okamoto A., Isonishi S., Ochiai K., Ohtake Y. (2001). A novel gene, CRR9, which was up-regulated in CDDP-resistant ovarian tumor cell line, was associated with apoptosis. Biochem. Biophys. Res. Commun..

[6] James M.A., Wen W.D., Wang Y.A., Byers L.A., Heymach J.V., Coombes K.R., Girard L., Minna J., You M. (2012). Functional Characterization of CLPTM1L as a Lung Cancer Risk Candidate Gene in the 5p15.33 Locus. PLoS ONE.

[7] Ni Z.H., Tao K., Chen G., Chen Q.G., Tang J.M., Luo X.M., Yin P.H., Tang J.H., Wang X.B.A. (2012). CLPTM1L Is Overexpressed in Lung Cancer and Associated with Apoptosis. PLoS ONE.

[8] Zhang M., Wu X., Lu W., Ge Y.K., Wang X., Cai Z.M., Wu S. (2015). Rs401681 polymorphism in TERT-CLPTM1L was associated with bladder cancer risk: A meta-analysis. Iran J. Basic. Med. Sci..

[9] Haiman C.A., Chen G.K., Vachon C.M., Canzian F., Dunning A., Millikan R.C., Wang X.S., Ademuyiwa F., Ahmed S., Ambrosone C.B. (2011). A common variant at the TERT-CLPTM1L locus is associated with estrogen receptor-negative breast cancer. Nat. Genet..

[10] Rafnar T., Sulem P., Stacey S.N., Geller F., Gudmundsson J., Sigurdsson A., Jakobsdottir M., Helgadottir H., Thorlacius S., Aben K.K.H. (2009). Sequence variants at the TERT-CLPTM1L locus associate with many cancer types. Nat. Genet..

[11] Petersen G.M., Amundadottir L., Fuchs C.S., Kraft P., Stolzenberg-Solomon R.Z., Jacobs K.B., Arslan A.A., Bueno-de-Mesquita H.B., Gallinger S., Gross M. (2010). A genome-wide association study identifies pancreatic cancer susceptibility loci on chromosomes 13q22.1, 1q32.1 and 5p15.33. Nat. Genet..

[12] Yin J., Li Y., Yin M., Sun J., Liu L., Qin Q., Li X., Long L., Nie S., Wei S. (2012). TERT-CLPTM1L polymorphism rs401681 contributes to cancers risk: Evidence from a meta-analysis based on 29 publications. PLoS ONE.

[13] Tian J., Wang Y., Dong Y., Chang J., Wu Y., Chang S., Che G. (2022). Cumulative Evidence for Relationships Between Multiple Variants in the TERT and CLPTM1L Region and Risk of Cancer and Non-Cancer Disease. Front. Oncol..

[14] Yoon S.-L., Jung S.-I., Do E.-J., Lee S.-R., Lee S.-Y., Chu I.-S., Kim W.-J., Jung J., Kim C.S., Cheon S.-H. (2010). Short rare hTERT-VNTR2-2nd alleles are associated with prostate cancer susceptibility and influence gene expression. BMC Cancer.

[15] Hofer P., Zerelles J., Baierl A., Madersbacher S., Schatzl G., Maj-Hes A., Sutterlüty-Fall H., Gsur A. (2013). MNS16A tandem repeat minisatellite of human telomerase gene and prostate cancer susceptibility. Mutagenesis.

[16] Kwon J.A., Jeong M.S., Yoon S.L., Mun J.Y., Kim M.H., Yang G.E., Park S.H., Chung J.W., Choi Y.H., Cha H.J. (2019). The hTERT-VNTR2-2(nd) alleles are involved in genomic stability in gastrointestinal cancer. Genes Genom..

[17] Diler S.B., Polat F., Bingöl G. (2019). The MNS16A VNTR polymorphism of the TERT gene in bladder cancer. Turk. J. Urol..

[18] Benson G. (1999). Tandem repeats finder: A program to analyze DNA sequences. Nucleic Acids Res..

[19] Seol S.-Y., Lee S.-Y., Kim Y.-D., Do E.-J., Kwon J.-A., Kim S.I., Chu I.-S., Leem S.-H. (2008). Minisatellite polymorphisms of the SLC6A19: Susceptibility in hypertension. Biochem. Biophys. Res. Commun..

[20] Yoon Y.-H., Seol S.-Y., Heo J., Chung C.-N., Park I.-H., Leem S.-H. (2008). Analysis of VNTRs in the Solute Carrier Family 6, Member 18 (SLC6A18) and Lack of Association with Hypertension. DNA Cell Biol..

[21] Yoon S.-L., Kim D.C., Cho S.H., Lee S.-Y., Chu I.-S., Heo J., Leem S.-H. (2010). Susceptibility for breast cancer in young patients with short rare minisatellite alleles of BORIS. BMB Rep..

[22] Nei M., Roychoudhury A.K.J.G. (1974). Sampling variances of heterozygosity and genetic distance. Genetics.

[23] Nakamura Y., Koyama K., Matsushima M. (1998). VNTR (variable number of tandem repeat) sequences as transcriptional, translational, or functional regulators. J. Human Genet..

[24] Greenwood T.A., Kelsoe J.R. (2003). Promoter and intronic variants affect the transcriptional regulation of the human dopamine transporter gene. Genomics.

[25] De Roeck A., Duchateau L., Van Dongen J., Cacace R., Bjerke M., Van den Bossche T., Cras P., Vandenberghe R., De Deyn P.P., Engelborghs S. (2018). An intronic VNTR affects splicing of ABCA7 and increases risk of Alzheimer’s disease. Acta Neuropathol..

[26] Brookes K.J. (2013). The VNTR in complex disorders: The forgotten polymorphisms? A functional way forward?. Genomics.

[27] Jeong Y.H., Kim M.C., Ahn E.K., Seol S.Y., Do E.J., Choi H.J., Chu I.S., Kim W.J., Kim W.J., Sunwoo Y. (2007). Rare Exonic Minisatellite Alleles in MUC2 Influence Susceptibility to Gastric Carcinoma. PLoS ONE.

[28] Kwon J.A., Lee S.Y., Ahn E.K., Seol S.Y., Kim M.C., Kim S.J., Kim S.I., Chu I.S., Leem S.H. (2010). Short Rare MUC6 Minisatellites-5 Alleles Influence Susceptibility to Gastric Carcinoma by Regulating Gene Expression. Hum. Mutat..

[29] Karami S., Han Y., Pande M., Cheng I., Rudd J., Pierce B.L., Nutter E.L., Schumacher F.R., Kote-Jarai Z., Lindstrom S. (2016). Telomere structure and maintenance gene variants and risk of five cancer types. Int. J. Cancer.

[30] Jia J.P., Bosley A.D., Thompson A., Hoskins J.W., Cheuk A., Collins I., Parikh H., Xiao Z., Ylaya K., Dzyadyk M. (2014). CLPTM1L Promotes Growth and Enhances Aneuploidy in Pancreatic Cancer Cells. Cancer Res..

[31] Puskas L.G., Man I., Szebeni G., Tiszlavicz L., Tsai S., James M.A. (2016). Novel Anti-CRR9/CLPTM1L Antibodies with Antitumorigenic Activity Inhibit Cell Surface Accumulation, PI3K Interaction, and Survival Signaling. Mol. Cancer Ther..

[32] Liao Y., Zou X., Wang K., Wang Y., Wang M., Guo T., Zhong B., Jiang N. (2021). Comprehensive analysis of Transcription Factors identified novel prognostic biomarker in human bladder cancer. J. Cancer.

[33] Gerber A.N., Newton R., Sasse S.K. (2021). Repression of transcription by the glucocorticoid receptor: A parsimonious model for the genomics era. J. Biol. Chem..

[34] McBeth L., Grabnar M., Selman S., Hinds T.D. (2015). Involvement of the Androgen and Glucocorticoid Receptors in Bladder Cancer. Int. J. Endocrinol..

[35] Yamashita H., Kawasawa Y.I., Shuman L., Zheng Z., Tran T., Walter V., Warrick J.I., Chen G., Al-Ahmadie H., Kaag M. (2019). Repression of transcription factor AP-2 α by PPARgamma reveals a novel transcriptional circuit in basal-squamous bladder cancer. Oncogenesis.

[36] McEwan I.J., Gustafsson J. (1997). Interaction of the human androgen receptor transactivation function with the general transcription factor TFIIF. Proc. Natl. Acad. Sci. USA.

[37] Martinez-Rojo E., Berumen L.C., Garcia-Alcocer G., Escobar-Cabrera J. (2021). The Role of Androgens and Androgen Receptor in Human Bladder Cancer. Biomolecules.

[38] Roy A.L. (2012). Biochemistry and biology of the inducible multifunctional transcription factor TFII-I: 10 years later. Gene.

[39] Sharron Lin X., Hu L., Sandy K., Correll M., Quackenbush J., Wu C.L., Scott McDougal W. (2014). Differentiating progressive from nonprogressive T1 bladder cancer by gene expression profiling: Applying RNA-sequencing analysis on archived specimens. Urol. Oncol..

[40] Kashiwagi E., Izumi H., Yasuniwa Y., Baba R., Doi Y., Kidani A., Arao T., Nishio K., Naito S., Kohno K. (2011). Enhanced expression of nuclear factor I/B in oxaliplatin-resistant human cancer cell lines. Cancer Sci..

[41] Adshead J.M., Ogden C.W., Penny M.A., Stuart E.T., Kessling A.M. (1999). The expression of PAX5 in human transitional cell carcinoma of the bladder: Relationship with de-differentiation. BJU Int..

[42] Esrig D., Elmajian D., Groshen S., Freeman J.A., Stein J.P., Chen S.C., Nichols P.W., Skinner D.G., Jones P.A., Cote R.J. (1994). Accumulation of nuclear p53 and tumor progression in bladder cancer. N. Engl. J. Med..

[43] Huh H.D., Kim D.H., Jeong H.S., Park H.W. (2019). Regulation of TEAD Transcription Factors in Cancer Biology. Cells.

[44] Zhu S., Yoon K., Sterneck E., Johnson P.F., Smart R.C. (2002). CCAAT/enhancer binding protein-β is a mediator of keratinocyte survival and skin tumorigenesis involving oncogenic Ras signaling. Proc. Natl. Acad. Sci. USA.

[45] Kiuru M., Busam K.J. (2017). The NF1 gene in tumor syndromes and melanoma. Lab. Investig..

[46] Kim W.G., Cheng S.Y. (2013). Thyroid hormone receptors and cancer. Biochim. Biophys. Acta.

[47] Liu Z., Zhu Y., Li F., Xie Y. (2020). GATA1-regulated JAG1 promotes ovarian cancer progression by activating Notch signal pathway. Protoplasma.

[48] Yu J., Liu M., Liu H., Zhou L. (2019). GATA1 promotes colorectal cancer cell proliferation, migration and invasion via activating AKT signaling pathway. Mol. Cell. Biochem..

