# Supplementary material for: Exploring the Relationship between CLPTM1L-MS2 Variants and Susceptibility to Bladder Cancer
Source: Genes (Basel). 2023 Dec 28;15(1):50. doi: 10.3390/genes15010050 (PMC10815179; doi:10.3390/genes15010050)

Table S1. Frequency of *CLPTM1L*-VNTR (MS1, MS3, MS4) alleles between controls and bladder cancer cases

TR	Size	Cancer-free controls		Bladder cancer		OR (95% CI)	*P*
		N=856	Frequency	N=340	Frequency		
MS1 TR							
12	866	3	0.0035	2	0.0059	1.68 (0.28-10.11)	0.5654
15	1058	849	0.9918	336	0.9882	0.69 (0.20-2.38)	0.5578
18	1250	1	0.0012	0	-	ND	0.5284
21	1442	1	0.0012	1	0.0029	2.52 (0.16-40.44)	0.4985
26	1762	2	0.0023	1	0.0029	1.26 (0.11-13.94)	0.8504
TR	Size	N=834	Frequency	N=342	Frequency	OR (95% CI)	*P*
MS3 TR							
76	2386	19	0.0228	11	0.0322	1.43 (0.67-3.03)	0.3541
94	2908	24	0.0288	13	0.0380	1.33 (0.67-2.65)	0.4100
96	2966	632	0.7578	254	0.7427	0.92 (0.69-1.23)	0.5853
98	3024	8	0.0096	2	0.0058	0.61 (0.13-2.87)	0.5254
100	3082	11	0.0132	5	0.0146	1.11 (0.38-3.22)	0.8475
102	3140	114	0.1367	47	0.1374	1.01 (0.70-1.45)	0.9734
104	3198	10	0.0120	2	0.0058	0.48 (0.11-2.22)	0.3412
106	3256	2	0.0024	1	0.0029	1.22 (0.11-13.50)	0.8710
109	3343	2	0.0024	3	0.0088	3.68 (0.61-22.13)	0.1271
112	3430	12	0.0144	3	0.0088	0.61 (0.17-2.16)	0.4357
128	3894	0	-	1	0.0029	ND	0.1182
TR	Size	N=864	Frequency	N=340	Frequency	OR (95% CI)	*P*
MS4 TR							
6	416	16	0.0185	12	0.0353	1.94 (0.91-4.14)	0.0821
8	530	848	0.9815	328	0.9647	0.52 (0.24-1.10)	

OR, odds ratio; CI, confidence internal; ND, not determined

Table S2. Frequency of *CLPTM1L*-VNTR (MS1, MS3, MS4) genotypes between controls and bladder cancer cases

Genotype	Cancer-free controls		Bladder cancer		OR (95% CI)	*P*
MS1	N=428	Frequency	N=170	Frequency		
12/15	3	0.0070	2	0.0118	1.69 (0.28-10.18)	0.5646
15/15	421	0.9836	166	0.9765	0.69 (0.20-2.39)	0.5559
15/18	1	0.0023	0	-	ND	0.5282
15/21	1	0.0023	1	0.0059	2.53 (0.16-40.63)	0.4981
15/26	2	0.0047	1	0.0059	1.26 (0.11-13.99)	0.8502
MS3	N=417	Frequency	N=171	Frequency	OR (95% CI)	*P*
76/76	3	0.0072	3	0.0175	2.46 (0.49-12.33)	0.2568
76/96	12	0.0288	4	0.0234	0.81 (0.26-2.54)	0.7155
76/102	0	-	1	0.0058	ND	0.1181
76/112	1	0.0024	0	-	ND	0.5216
94/94	11	0.0264	6	0.0351	1.34 (0.49-3.69)	0.5671
94/100	1	0.0024	0	-	ND	0.5216
94/102	1	0.0024	1	0.0058	2.45 (0.15-39.35)	0.5141
96/96	256	0.6139	104	0.6082	0.98 (0.68-1.41)	0.8971
96/98	8	0.0192	2	0.0117	0.61 (0.13-2.88)	0.5236
96/100	7	0.0168	3	0.0175	1.05 (0.27-4.09)	0.9486
96/102	72	0.1727	29	0.1696	0.98 (0.61-1.57)	0.9286
96/104	9	0.0216	2	0.0117	0.54 (0.11-2.51)	0.4216
96/106	1	0.0024	1	0.0058	2.45 (0.15-39.35)	0.5141
96/109	1	0.0024	1	0.0058	2.45 (0.15-39.35)	0.5141
96/112	10	0.0240	3	0.0175	0.73 (0.20-2.67)	0.6297
96/128	0	-	1	0.0058	ND	0.1181
100/100	0	-	1	0.0058	ND	0.1181
100/102	3	0.0072	0	-	ND	0.2661
102/102	18	0.0432	8	0.0468	1.09 (0.46-2.55)	0.8463
102/109	1	0.0024	0	-	ND	0.5216
102/112	1	0.0024	0	-	ND	0.5216
104/106	1	0.0024	0	-	ND	0.5216
109/109	0	-	1	0.0058	ND	0.1181
MS4	N=432	Frequency	N=170	Frequency	OR (95% CI)	*P*
6/8	16	0.0370	12	0.0706	1.97 (0.91-4.27)	0.0785
8/8	416	0.9630	158	0.9294	0.51 (0.23-1.09)	

OR, odds ratio; CI, confidence internal; ND, not determined

Table S3. Composition of repeat units in most common alleles of *CLPTM1L*-MS2

**Repeat unit**	**Sequences**
Consensus	TGAGGCCCTGTCCACCAAGCAGAGAGTCC
MS2-1	TGAGGC**T**CTGTCCACCAAGCAG**TGGT**GAGTCC
MS2-2	TGAGGCCCTGTCCACCAAGCAGA**T**AGTCC
MS2-3	TGAGGCCCTGTCCACCAAGCAGAGAGTCC
MS2-4	TGAGGC**T**CTGTCCACCAAGCAGAGAGTCC
MS2-5	TGAGGC**T**CTGTCCACCA**G**GCAGAGAGTCC
MS2-6	TGAGGCCCTGTCCACCA**G**GCAGAGAGTCC
MS2-7	TGAGGCCCTGTCCACCAAGCAG**TGGT**GAGTCC
MS2-8	TGAGGCCCTGTCCACCAAGCAGAG**GCC**
**MS2-TR**	**Composition of repeat units in most common alleles**
MS2-23TR	1-2-1-3-3-4-4-3-3-5-6-4-6-6-6-6-3-3-4-4-7-8-△

△: 0.8 repeat

Table S4. Analysis of identities between repeat units in *CLPTM1L*-MS2

**Identities (%)**	**MS2-1**	**MS2-2**	**MS2-3**	**MS2-4**	**MS2-5**	**MS2-6**	**MS2-7**	**MS2-8**
MS2-1	100							
MS2-2	81	100						
MS2-3	84	97	100					
MS2-4	88	93	97	100				
MS2-5	84	90	93	97	100			
MS2-6	81	93	97	93	97	100		
MS2-7	97	84	88	84	81	84	100	
MS2-8	78	90	90	86	83	86	81	100

Supplementary Figure S1. Composition of putative transcriptional factors on most common alleles of *CLPTM1L*-MS2

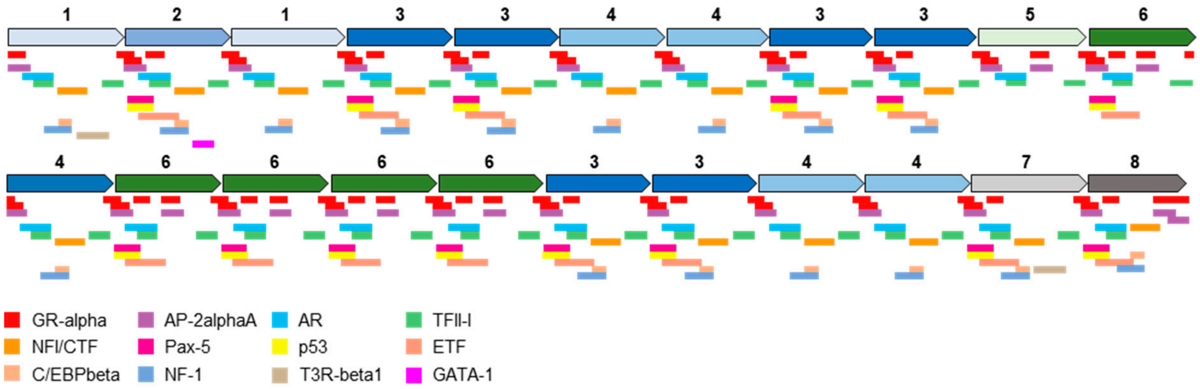

Supplement: Supplementary file 1 [file genes-15-00050-s001.zip › genes-2731875-supplementary.pdf]
